# Crystal structure of (2*E*)-3-(3-eth­oxy-4-hy­droxy­phen­yl)-1-(4-hy­droxy­phen­yl)prop-2-en-1-one

**DOI:** 10.1107/S1600536814023368

**Published:** 2014-10-29

**Authors:** R. Vasanthi, D. Reuben Jonathan, K. S. Elizhlarasi, B. K. Revathi, G. Usha

**Affiliations:** a PG and Research Department of Physics, Queen Mary’s College, Chennai-4, Tamilnadu, India; b Department of Chemistry, Madras Christian College, Chennai-59, India

**Keywords:** crystal structure, prop-2-en-1-one, hydrogen bonding,

## Abstract

In the title compound, C_17_H_16_O_4_, the dihedral angle between the benzene rings is 21.22 (1)° and the mean plane of the prop-2-en-1-one group makes dihedral angles of 10.60 (1) and 11.28 (1)°, respectively, with those of the hy­droxy­phenyl and eth­oxy­phenyl rings. The eth­oxy substituent forms a dihedral angle of 88.79 (2)° with the the prop-2-en-1-one group, which is found to be slightly twisted. In the crystal, phenolic O—H⋯O hydrogen bonds to the carbonyl O atom form a two-dimensional supra­molecular network structure lying parallel to (010).

## Related literature   

For the biological activity of chalcone derivatives, see: Nowakowska (2007 ▶); Ram *et al.* (2000 ▶); Khatib *et al.* (2005 ▶); Papo & Shai (2003 ▶). For related structures, see: Jasinski *et al.* (2011 ▶); Sathya *et al.* (2014 ▶); Joothamongkhon *et al.* (2010 ▶); Horkaew *et al.* (2010 ▶). For the synthesis, see: Sidharthan *et al.* (2012 ▶); Chitra *et al.* (2013 ▶); Sathya *et al.* (2014 ▶).
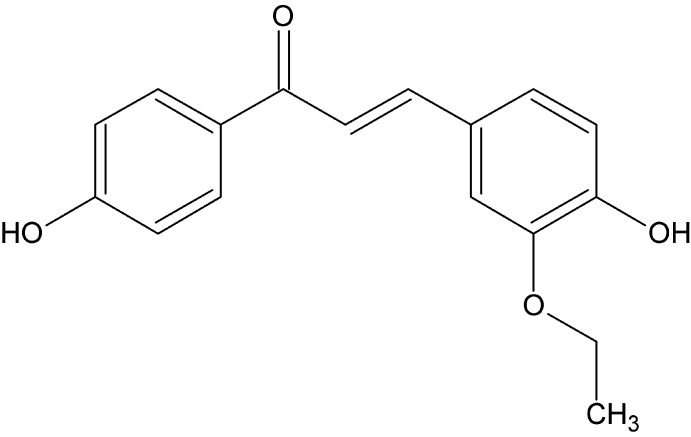


## Experimental   

### Crystal data  

C_17_H_16_O_4_
*M* *_r_* = 284.30
Orthorhombic, 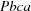

*a* = 16.3670 (4) Å
*b* = 10.5512 (3) Å
*c* = 16.6153 (4) Å
*V* = 2869.32 (13) Å^3^
*Z* = 8
Mo *K*α radiation
μ = 0.09 mm^−1^
*T* = 293 K
0.22 × 0.21 × 0.19 mm

### Data collection  

Bruker Kappa APEXII CCD diffractometer
Absorption correction: multi-scan (*SADABS*; Bruker, 2004 ▶) *T*_min_ = 0.970, *T*_max_ = 0.985
14416 measured reflections
3592 independent reflections
2619 reflections with *I* > 2σ(*I*)
*R*_int_ = 0.028

### Refinement  

*R*[*F* ^2^ > 2σ(*F* ^2^)] = 0.058
*wR*(*F* ^2^) = 0.215
*S* = 0.72
3592 reflections
190 parameters
H-atom parameters constrained
Δρ_max_ = 0.74 e Å^−3^
Δρ_min_ = −0.43 e Å^−3^

### 

Data collection: *APEX2* (Bruker, 2004 ▶); cell refinement: *APEX2* and *SAINT* (Bruker, 2004 ▶); data reduction: *SAINT* and *XPREP* (Bruker, 2004 ▶); program(s) used to solve structure: *SHELXS97* (Sheldrick, 2008 ▶); program(s) used to refine structure: *SHELXL97* (Sheldrick, 2008 ▶); molecular graphics: *ORTEP-3 for Windows* (Farrugia, 2012 ▶); software used to prepare material for publication: *SHELXL97*.

## Supplementary Material

Crystal structure: contains datablock(s) I, New_Global_Publ_Block. DOI: 10.1107/S1600536814023368/zs2316sup1.cif

Structure factors: contains datablock(s) I. DOI: 10.1107/S1600536814023368/zs2316Isup2.hkl

Supporting information file. DOI: 10.1107/S1600536814023368/zs2316Isup3.cml

**Fig. 1.**
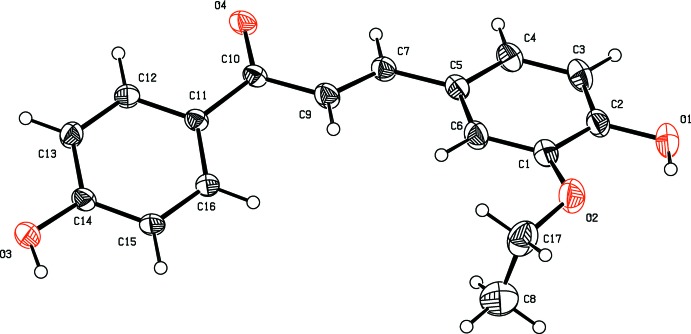
The molecular structure of the title compound, with displacement ellipsoids drawn at the 30% probability level.

. DOI: 10.1107/S1600536814023368/zs2316fig1.tif
The mol­ecular structure of the title compound, with displacement ellipsoids drawn at the 30% probability level.

**Fig. 2.**
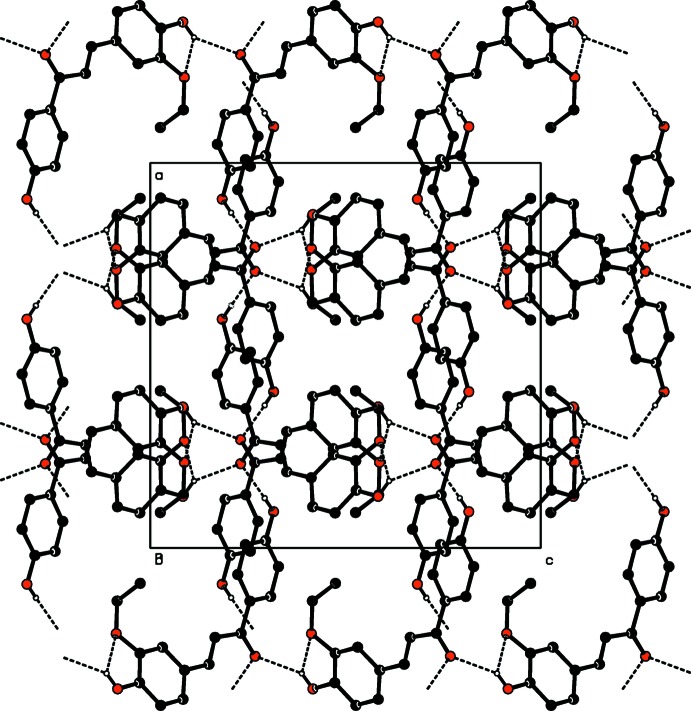
The packing of the molecules in the unit cell. Non-associative H-atoms are omitted and dashed lines indicate hydrogen bonds.

. DOI: 10.1107/S1600536814023368/zs2316fig2.tif
The packing of the mol­ecules in the unit cell. Non-associative H-atoms are omitted and dashed lines indicate hydrogen bonds.

CCDC reference: 1030607

Additional supporting information:  crystallographic information; 3D view; checkCIF report

## Figures and Tables

**Table 1:** Hydrogen-bond geometry (, )

*D*H*A*	*D*H	H*A*	*D* *A*	*D*H*A*
O1H1O4^i^	0.82	2.25	2.958(2)	145
O3H3*A*O4^ii^	0.82	1.95	2.766(2)	171

Symmetry codes: (i) 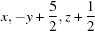; (ii) 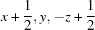.

## supplementary crystallographic information

### S1. Comment

Chalcones belonging to the flavonoid family constitute an important group of natural products due to their unforseen pharmacological potential. Chemically they consist of open-chain flavonoids in which the two aromatic rings are joined by a three-carbon α,β-unsaturated carbonyl system. The radical quenching properties of the phenolic groups present in many chalcones have raised interest in using the compounds or chalcone-rich plant extracts as drugs or food preservatives. Chalcones have been reported to possess many exciting activities which include anti-inflammatory, antimicrobial, antifungal, antioxidant, cytotoxic, anti-tumor and anticancer (Nowakowska, 2007). A number of chalcones having hydroxy or alkoxy groups in different position have been observed to possess vasodilatory (Ram *et al.*, 2000), antimitotic (Khatib *et al.*, 2005) and antimalarial activities (Papo & Shai, 2003). The crystal structures of closely related chalcones, *viz*., (*E*)-3-(4-ethoxyphenyl)-1-(2-hydroxyphenyl)prop-2-en-one (Horkaew *et al.*, 2010) (*Z*)-3-(anthracen-9-yl)-1-(2-ethoxyphenyl)prop-2-en-one (Joothamongkhon *et al.*, 2010) have been reported. The enormous research potentials of this group of compounds prompted us to synthesize an analogous compound, the chalcone derivative C_17_H_16_O_4_ and we report the crystal structure in this communication.

In the title compound, the C—C bond lengths of the hydroxyphenyl and ethoxyphenyl rings are in the range of 1.376 (3)–1.398 (3)Å and 1.366 (3)–1.408 (3) Å, respectively, and are in good agreement with similar reported values [1.374 (3)–1.389 (3) Å and 1.369 (3)–1.401 (3) Å] (Sathya *et al.*, 2014). The C—O bond lengths 1.354 (2)–1.450 (3)Å and 1.237 (2)Å, respectively, indicate the single and double bond characters and are comparable with literature values. The bond angles C9—C10—C11 [118.40 (15)°], C5—C7—C9 [127.10 (19)°] are comparable with those in similar reported structure (Sathya *et al.*, 2014; Jasinski *et al.*, 2011). The prop-2-en-1-one group is twisted slightly with O4—C10—C11—C12 and C7—C9—C10—O4 torsion angles of -10.8 (3) and -14.2 (3)°, respectively, and are comparable with those in similar reported structures (Sathya *et al.*, 2014; Jasinski *et al.*, 2011). The torsion angle C1—O2—C17—C1 [76.2 (3)°] indicates that the ethoxy group is in a +*synclinal* (+*sc*) orientation with respect to the benzene ring. The dihedral angle between the benzene rings is 21.22 (1)°. The prop-2-en-one group makes dihedral angles of 10.60 (1) and 11.28 (1)° with the hydroxyphenyl and ethoxyphenyl rings, respectively. The ethoxy substituent forms a dihedral angle of 88.79 (2)° with the prop-2-en-one group. The molecular conformation is stabilized by interamolecular O1—H···O2 and C7—H···O4 interactions (Table 1).

In the crystal packing (Fig. 2), the molecule are linked through hydroxyl O1—H···O4^i^ and O3—H···O4^ii^ hydrogen bonds to the carbonyl O-atom acceptor (Table 1). Atom O4 acts as a tricentre being involved also in the previously mentioned intramolecular interaction with C7—H7. The overall structure is a two-dimensional supramolecular network lying parallel to (010).

### S2. Experimental

This is the acid catalyzed Claisen-Schmidt reaction and the procedure (Sidharthan *et al.*, 2012; Chitra *et al.*, 2013; Sathya *et al.*, 2014) adopted in the synthesis of the typical chalcone diol is represented here. Dry HCl gas was passed for one hour through a well cooled and stirred solution of 4-hydroxyacetophenone (0.05 mol) and 4-hydroxy-3-ethoxybenzaldehyde (0.05 mol) in 120 mL of absolute alcohol in a 250 mL round-bottomed flask. A wine red coloured solution was formed. On addition of a sufficient quantity of ice cold water, a yellow precipitate of (2*E*)-3-(4-hydroxy-3-ethoxyphenyl)-1-(4-hydroxyphenyl)prop-2-en -1-one was formed, which was filtered, then washed with double distilled water and finally allowed to dry. The dried product was re-crystallized from hot ethanol: yield 80%.

### S3. Refinement

Hydrogen atoms were positioned geometrically and treated as riding on their parent atoms, with C—H distances of 0.93 Å, O—H distances of 0.82 Å with *U*_iso_(H)= 1.5 *U*_eq_(c-methyl) and *U*_iso_(H)= 1.2Ueq(C) for other H atom.

### Crystal data

**Table:**

C_17_H_16_O_4_	*F*(000) = 1200
*M**_r_* = 284.30	*D*_x_ = 1.316 Mg m^−^^3^
Orthorhombic, *P**b**c**a*	Mo *K*α radiation, λ = 0.71073 Å
Hall symbol: -P 2ac 2ab	Cell parameters from 3592 reflections
*a* = 16.3670 (4) Å	θ = 2.5–28.4°
*b* = 10.5512 (3) Å	µ = 0.09 mm^−^^1^
*c* = 16.6153 (4) Å	*T* = 293 K
*V* = 2869.32 (13) Å^3^	Block, colourless
*Z* = 8	0.22 × 0.21 × 0.19 mm

### Data collection

**Table:**

Bruker Kappa APEXII CCD diffractometer	3592 independent reflections
Radiation source: fine-focus sealed tube	2619 reflections with *I* > 2σ(*I*)
Graphite monochromator	*R*_int_ = 0.028
ω and φ scan	θ_max_ = 28.4°, θ_min_ = 2.5°
Absorption correction: multi-scan (*SADABS*; Bruker, 2004)	*h* = −21→21
*T*_min_ = 0.970, *T*_max_ = 0.985	*k* = −11→14
14416 measured reflections	*l* = −21→22

### Refinement

**Table:**

Refinement on *F*^2^	Primary atom site location: structure-invariant direct methods
Least-squares matrix: full	Secondary atom site location: difference Fourier map
*R*[*F*^2^ > 2σ(*F*^2^)] = 0.058	Hydrogen site location: inferred from neighbouring sites
*wR*(*F*^2^) = 0.215	H-atom parameters constrained
*S* = 0.72	*w* = 1/[σ^2^(*F*_o_^2^) + (0.189*P*)^2^ + 2.3286*P*] where *P* = (*F*_o_^2^ + 2*F*_c_^2^)/3
3592 reflections	(Δ/σ)_max_ < 0.001
190 parameters	Δρ_max_ = 0.74 e Å^−^^3^
0 restraints	Δρ_min_ = −0.43 e Å^−^^3^

### Special details

**Table:**

Geometry. All esds (except the esd in the dihedral angle between two l.s. planes) are estimated using the full covariance matrix. The cell esds are taken into account individually in the estimation of esds in distances, angles and torsion angles; correlations between esds in cell parameters are only used when they are defined by crystal symmetry. An approximate (isotropic) treatment of cell esds is used for estimating esds involving l.s. planes.
Refinement. Refinement of F^2^ against ALL reflections. The weighted R-factor wR and goodness of fit S are based on F^2^, conventional R-factors R are based on F, with F set to zero for negative F^2^. The threshold expression of F^2^ > 2sigma(F^2^) is used only for calculating R-factors(gt) etc. and is not relevant to the choice of reflections for refinement. R-factors based on F^2^ are statistically about twice as large as those based on F, and R- factors based on ALL data will be even larger.

### Fractional atomic coordinates and isotropic or equivalent isotropic displacement parameters (Å^2^)

**Table:**

	*x*	*y*	*z*	*U*_iso_*/*U*_eq_	
C8	0.4080 (2)	1.4090 (4)	0.5257 (2)	0.0948 (11)	
H8A	0.4638	1.3812	0.5246	0.142*	
H8B	0.4060	1.4969	0.5404	0.142*	
H8C	0.3841	1.3980	0.4734	0.142*	
C1	0.22651 (13)	1.3374 (2)	0.52630 (13)	0.0481 (5)	
C2	0.14997 (13)	1.39834 (19)	0.52749 (12)	0.0459 (5)	
C3	0.09113 (13)	1.3644 (2)	0.47328 (13)	0.0519 (5)	
H3	0.0402	1.4036	0.4748	0.062*	
C4	0.10695 (12)	1.2720 (2)	0.41605 (12)	0.0464 (5)	
H4	0.0662	1.2491	0.3798	0.056*	
C5	0.18244 (11)	1.21328 (18)	0.41214 (11)	0.0405 (4)	
C6	0.24215 (12)	1.2466 (2)	0.46881 (13)	0.0491 (5)	
H6	0.2929	1.2069	0.4676	0.059*	
C7	0.19783 (11)	1.11972 (18)	0.34958 (11)	0.0405 (4)	
H7	0.1538	1.0987	0.3169	0.049*	
C9	0.26765 (11)	1.0613 (2)	0.33418 (12)	0.0424 (4)	
H9	0.3122	1.0779	0.3673	0.051*	
C10	0.27837 (10)	0.97178 (17)	0.26748 (11)	0.0353 (4)	
C11	0.36197 (10)	0.93496 (16)	0.24468 (10)	0.0331 (4)	
C12	0.37395 (11)	0.83378 (18)	0.19164 (12)	0.0404 (4)	
H12	0.3289	0.7928	0.1695	0.048*	
C13	0.45150 (11)	0.79391 (19)	0.17169 (12)	0.0439 (5)	
H13	0.4586	0.7265	0.1363	0.053*	
C14	0.51931 (10)	0.85457 (17)	0.20461 (11)	0.0369 (4)	
C15	0.50858 (11)	0.95616 (18)	0.25631 (12)	0.0388 (4)	
H15	0.5537	0.9978	0.2777	0.047*	
C16	0.43087 (11)	0.99521 (17)	0.27588 (11)	0.0383 (4)	
H16	0.4241	1.0634	0.3107	0.046*	
C17	0.36220 (17)	1.3339 (3)	0.58491 (17)	0.0685 (7)	
H17A	0.3649	1.2449	0.5706	0.082*	
H17B	0.3866	1.3443	0.6377	0.082*	
O1	0.13388 (11)	1.48915 (16)	0.58349 (10)	0.0614 (5)	
H1	0.1742	1.4996	0.6120	0.092*	
O2	0.27758 (11)	1.3744 (2)	0.58724 (11)	0.0693 (5)	
O3	0.59416 (8)	0.81036 (15)	0.18410 (10)	0.0521 (4)	
H3A	0.6294	0.8522	0.2071	0.078*	
O4	0.21879 (8)	0.92718 (14)	0.23142 (9)	0.0459 (4)	

### Atomic displacement parameters (Å^2^)

**Table:**

	*U*^11^	*U*^22^	*U*^33^	*U*^12^	*U*^13^	*U*^23^
C8	0.073 (2)	0.102 (3)	0.109 (3)	−0.0156 (18)	0.0008 (18)	−0.020 (2)
C1	0.0428 (10)	0.0523 (11)	0.0490 (11)	0.0000 (8)	0.0078 (8)	−0.0092 (8)
C2	0.0484 (11)	0.0444 (9)	0.0447 (10)	0.0038 (8)	0.0164 (8)	0.0003 (8)
C3	0.0447 (11)	0.0565 (12)	0.0546 (12)	0.0179 (9)	0.0094 (9)	0.0036 (9)
C4	0.0383 (10)	0.0543 (11)	0.0468 (11)	0.0080 (8)	0.0016 (8)	0.0021 (9)
C5	0.0352 (9)	0.0435 (9)	0.0430 (10)	0.0025 (7)	0.0056 (7)	0.0001 (7)
C6	0.0351 (9)	0.0587 (12)	0.0536 (12)	0.0053 (8)	0.0009 (8)	−0.0135 (9)
C7	0.0327 (8)	0.0458 (9)	0.0429 (10)	0.0003 (7)	−0.0004 (7)	−0.0022 (7)
C9	0.0291 (8)	0.0559 (11)	0.0423 (10)	0.0000 (7)	−0.0010 (7)	−0.0087 (8)
C10	0.0264 (8)	0.0425 (9)	0.0370 (9)	−0.0010 (6)	0.0000 (6)	0.0016 (7)
C11	0.0265 (8)	0.0395 (8)	0.0334 (8)	−0.0017 (6)	−0.0007 (6)	−0.0007 (6)
C12	0.0300 (8)	0.0465 (10)	0.0447 (10)	−0.0037 (7)	−0.0052 (7)	−0.0093 (7)
C13	0.0347 (9)	0.0460 (9)	0.0510 (11)	0.0021 (7)	−0.0030 (7)	−0.0160 (8)
C14	0.0266 (8)	0.0411 (8)	0.0428 (9)	0.0009 (6)	0.0008 (6)	0.0003 (7)
C15	0.0262 (8)	0.0459 (9)	0.0444 (10)	−0.0062 (7)	−0.0030 (7)	−0.0063 (7)
C16	0.0302 (9)	0.0414 (9)	0.0433 (10)	−0.0036 (7)	0.0007 (7)	−0.0081 (7)
C17	0.0640 (16)	0.0714 (16)	0.0699 (16)	0.0077 (12)	−0.0186 (12)	−0.0210 (13)
O1	0.0625 (10)	0.0616 (9)	0.0600 (10)	0.0136 (8)	0.0132 (8)	−0.0155 (7)
O2	0.0556 (10)	0.0857 (12)	0.0666 (11)	0.0044 (9)	−0.0012 (8)	−0.0341 (10)
O3	0.0292 (7)	0.0553 (8)	0.0719 (10)	0.0039 (6)	0.0018 (6)	−0.0147 (7)
O4	0.0266 (6)	0.0603 (9)	0.0508 (8)	−0.0045 (6)	−0.0024 (5)	−0.0078 (6)

### Geometric parameters (Å, º)

**Table:**

C8—C17	1.469 (5)	C9—H9	0.9300
C8—H8A	0.9600	C10—O4	1.237 (2)
C8—H8B	0.9600	C10—C11	1.472 (2)
C8—H8C	0.9600	C11—C16	1.394 (2)
C1—O2	1.370 (3)	C11—C12	1.398 (2)
C1—C6	1.377 (3)	C12—C13	1.378 (3)
C1—C2	1.408 (3)	C12—H12	0.9300
C2—O1	1.361 (2)	C13—C14	1.393 (3)
C2—C3	1.366 (3)	C13—H13	0.9300
C3—C4	1.387 (3)	C14—O3	1.354 (2)
C3—H3	0.9300	C14—C15	1.385 (3)
C4—C5	1.384 (3)	C15—C16	1.376 (3)
C4—H4	0.9300	C15—H15	0.9300
C5—C6	1.402 (3)	C16—H16	0.9300
C5—C7	1.455 (3)	C17—O2	1.450 (3)
C6—H6	0.9300	C17—H17A	0.9700
C7—C9	1.323 (3)	C17—H17B	0.9700
C7—H7	0.9300	O1—H1	0.8200
C9—C10	1.467 (3)	O3—H3A	0.8200
			
C17—C8—H8A	109.5	O4—C10—C9	121.10 (16)
C17—C8—H8B	109.5	O4—C10—C11	120.50 (16)
H8A—C8—H8B	109.5	C9—C10—C11	118.40 (15)
C17—C8—H8C	109.5	C16—C11—C12	117.97 (16)
H8A—C8—H8C	109.5	C16—C11—C10	122.39 (16)
H8B—C8—H8C	109.5	C12—C11—C10	119.61 (15)
O2—C1—C6	126.71 (19)	C13—C12—C11	120.93 (16)
O2—C1—C2	113.72 (18)	C13—C12—H12	119.5
C6—C1—C2	119.5 (2)	C11—C12—H12	119.5
O1—C2—C3	119.90 (19)	C12—C13—C14	119.96 (17)
O1—C2—C1	120.2 (2)	C12—C13—H13	120.0
C3—C2—C1	119.88 (18)	C14—C13—H13	120.0
C2—C3—C4	120.31 (19)	O3—C14—C15	122.50 (16)
C2—C3—H3	119.8	O3—C14—C13	117.62 (17)
C4—C3—H3	119.8	C15—C14—C13	119.88 (16)
C5—C4—C3	120.91 (19)	C16—C15—C14	119.71 (16)
C5—C4—H4	119.5	C16—C15—H15	120.1
C3—C4—H4	119.5	C14—C15—H15	120.1
C4—C5—C6	118.62 (18)	C15—C16—C11	121.54 (16)
C4—C5—C7	119.44 (18)	C15—C16—H16	119.2
C6—C5—C7	121.93 (17)	C11—C16—H16	119.2
C1—C6—C5	120.71 (19)	O2—C17—C8	110.3 (3)
C1—C6—H6	119.6	O2—C17—H17A	109.6
C5—C6—H6	119.6	C8—C17—H17A	109.6
C9—C7—C5	127.10 (18)	O2—C17—H17B	109.6
C9—C7—H7	116.4	C8—C17—H17B	109.6
C5—C7—H7	116.4	H17A—C17—H17B	108.1
C7—C9—C10	123.31 (17)	C2—O1—H1	109.5
C7—C9—H9	118.3	C1—O2—C17	118.63 (17)
C10—C9—H9	118.3	C14—O3—H3A	109.5
			
O2—C1—C2—O1	2.9 (3)	O4—C10—C11—C16	171.05 (18)
C6—C1—C2—O1	−179.4 (2)	C9—C10—C11—C16	−9.5 (3)
O2—C1—C2—C3	−175.8 (2)	O4—C10—C11—C12	−10.8 (3)
C6—C1—C2—C3	1.9 (3)	C9—C10—C11—C12	168.66 (18)
O1—C2—C3—C4	−179.95 (19)	C16—C11—C12—C13	0.8 (3)
C1—C2—C3—C4	−1.3 (3)	C10—C11—C12—C13	−177.40 (18)
C2—C3—C4—C5	−0.6 (3)	C11—C12—C13—C14	0.1 (3)
C3—C4—C5—C6	1.8 (3)	C12—C13—C14—O3	178.93 (19)
C3—C4—C5—C7	−177.92 (19)	C12—C13—C14—C15	−1.1 (3)
O2—C1—C6—C5	176.6 (2)	O3—C14—C15—C16	−178.90 (18)
C2—C1—C6—C5	−0.7 (3)	C13—C14—C15—C16	1.1 (3)
C4—C5—C6—C1	−1.1 (3)	C14—C15—C16—C11	−0.1 (3)
C7—C5—C6—C1	178.6 (2)	C12—C11—C16—C15	−0.8 (3)
C4—C5—C7—C9	175.3 (2)	C10—C11—C16—C15	177.37 (18)
C6—C5—C7—C9	−4.5 (3)	C6—C1—O2—C17	12.3 (4)
C5—C7—C9—C10	−177.43 (18)	C2—C1—O2—C17	−170.2 (2)
C7—C9—C10—O4	−14.2 (3)	C8—C17—O2—C1	76.2 (3)
C7—C9—C10—C11	166.32 (19)		

### Hydrogen-bond geometry (Å, º)

**Table:**

*D*—H···*A*	*D*—H	H···*A*	*D*···*A*	*D*—H···*A*
O1—H1···O4^i^	0.82	2.25	2.958 (2)	145
O3—H3*A*···O4^ii^	0.82	1.95	2.766 (2)	171
C7—H7···O4	0.93	2.53	2.846 (2)	100

Symmetry codes: (i) *x*, −*y*+5/2, *z*+1/2; (ii) *x*+1/2, *y*, −*z*+1/2.
