# Supplementary figures and images for: Crystal structure of (2E)-3-(3-eth­oxy-4-hy­droxy­phen­yl)-1-(4-hy­droxy­phen­yl)prop-2-en-1-one
Source: Acta Crystallogr Sect E Struct Rep Online. 2014 Oct 29;70(Pt 11):o1202–3. doi: 10.1107/S1600536814023368 (PMC4257333; doi:10.1107/S1600536814023368)

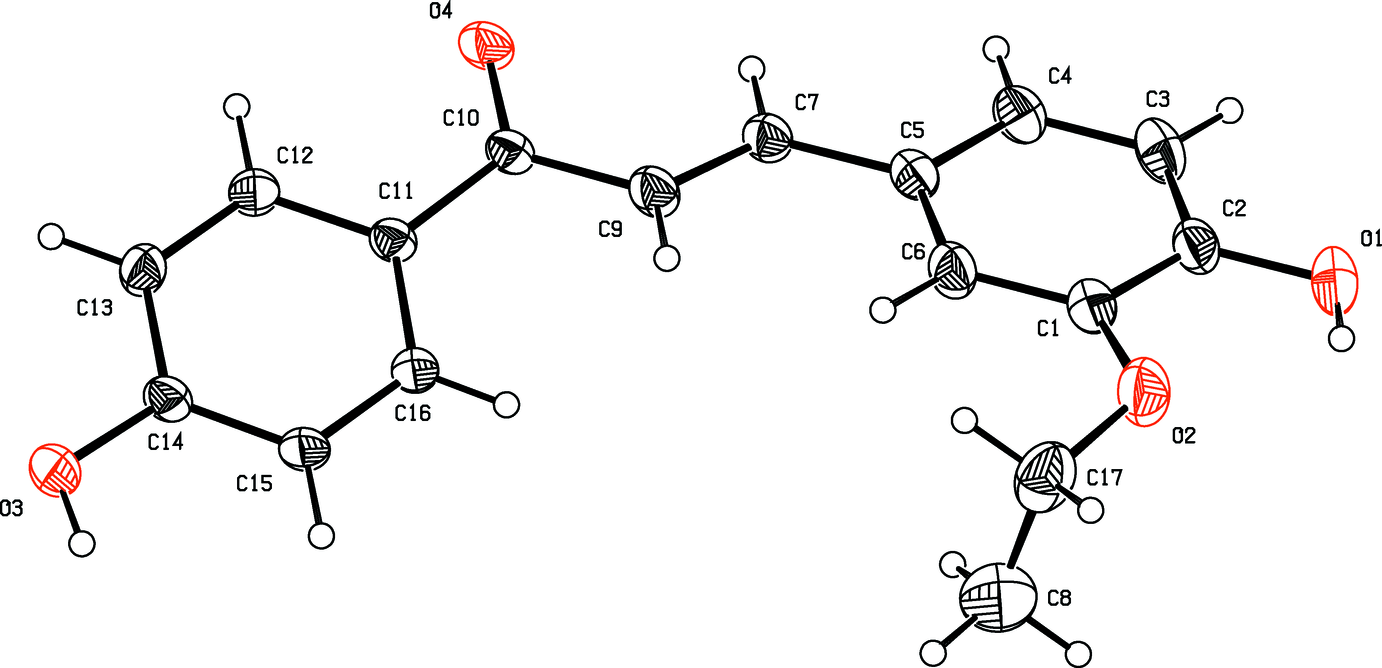

Supplement: Supplementary file 4 [file e-70-o1202-fig1.tif]

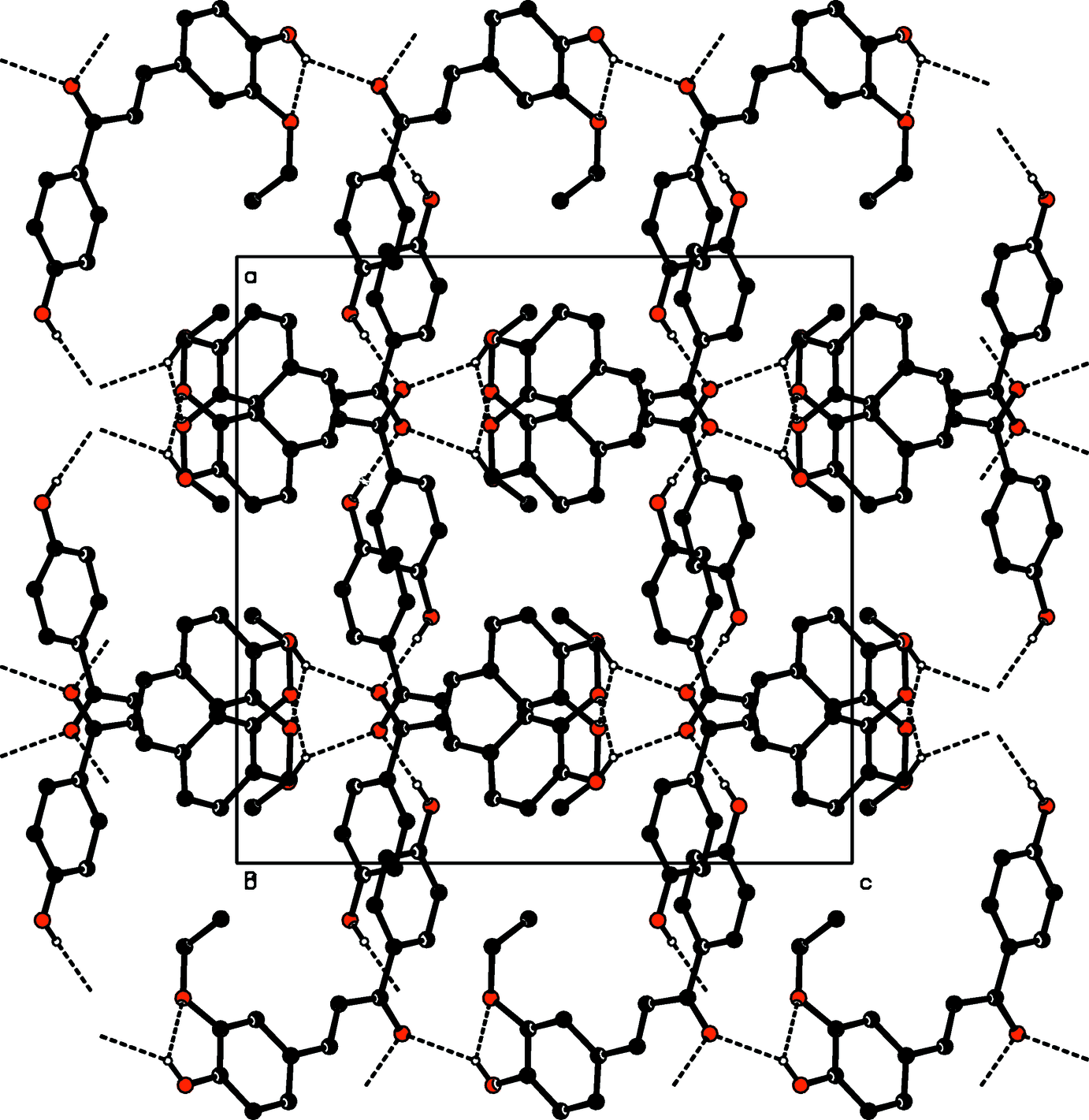

Supplement: Supplementary file 5 [file e-70-o1202-fig2.tif]
